# Hospital Meetings

**Published:** 1907-07-20

**Authors:** 


### HOSPITAL MEETINGS.

### HAMPSTEAD GENERAL HOSPITAL.

### The Proposed Medical Staff Change.—Amalgamation Question.—Subscribers' Committee Appointed.

An extraordinary general meeting of the subscribers of the Hampstead General Hospital was held at the Hospital, Haverstock Hill, on Tuesday evening, July 16, to enable the Council to lay before the subscribers "the arrangements which have been made for amalgamation with the North-West London Hospital," and to consider the following resolutions (1) "That the practice hitherto followed of appointing acting medical officers from among the members of the medical profession practising in Hampstead be continued," and (2) "That the number of physicians and surgeons on the consulting staff be augmented."

Mr. Edward Bond presided, and explained what would be the procedure of the meeting, and added that the occasion was a very grave one, and that the future interests of the hospital might prove to be very vitally bound up in the decision at which the meeting might arrive.

Mr. Ernest Collins (Chairman of the Council) said he had been asked to lay before the meeting the arrangements which had been come to with the King's Fund and the North-West London Hospital as regarded amalgamation. The Executive Committee of the King's Fund, in a letter dated February 7, 1906, after intimating their intention to recommend a grant of £5,000 to the Building Fund, expressed the opinion that the time had come when the hospital "should secure the services of an honorary medical staff of consulting surgeons and physicians, in accordance with the general practice of other hospitals of like importance." The Council considered very seriously what should be done, the general feeling of the subscribers being that nothing should be done which would be unjust to the present medical staff, who had served the hospital so faithfully and well for so many years. (Hear, hear.) Some twelve months later the King's Fund approached the Council again with a view to amalgamation with the North-West London Hospital, but the Council decided that they would settle the point as to the status of the medical staff before approaching the amalgamation question. Many conferences were held, the King's Fund in the first instance wishing that the present medical staff should be turned out at once and consultants appointed, a proposition which the Council refused to agree to. Eventually he got the King's Fund to agree that the present staff should stay for three years, and later it was decided that certain new regulations should be made as regarded the staff, the effect of which was, as regards the present senior members of the staff, that the service of two of their number would expire in five years, and two others in seven years, and the two junior members, who were appointed in September 1905, in thirteen years; the acting medical staff being composed eventually of consultants—four consultants (two surgeons and two physicians) to be appointed to the acting staff so soon as the extension buildings were ready for occupation. In the first instance the King's Fund assumed that they might appoint the consultants, but the Council resisted that, though not objecting to a consultation with the King's Fund in the nomination of the first three, and that was agreed to. The King's Fund would give them £1,500 a year, £2,000 towards the out-patients' department, free rent, and a large amount of moral support. The North-West London Hospital would hand over investments, etc., £5,500; stock-in-trade worth about £400; £500 belonging to the Samaritan Fund to be handed over to the trustees for the benefit of the Samaritan Fund of the combined hospitals. The investments, transferred into trustee securities, would yield some £170 a year. Then, if they wrote off, say, two-thirds of the present subscriptions and donations of £1,500 as a result of the amalgamation, that would give them £500 as the amount they would be likely to receive. Making a very ample allowance for the expenses which would be incurred in carrying on the extra out-patients' department, he calculated that they would receive for the benefit of the hospital about £1,500 a year surplus.

The Mayor of Hampstead (Councillor C. Hendrick) then moved the resolutions contained in the notice. The changes proposed were radical—indeed, almost revolutionary. (Hear, hear.) The Hospital was founded by a general practitioner for Hampstead people, and with the object of its being staffed, as it had been up to the present, by Hampstead medical men. It was now proposed to dispense sooner or later with the services of Hampstead medical men, and to include Kentish Town in the benefits that accrued from the work of the Hospital. These proposals affected the constitution of the Hospital fundamentally, and such a step should not have been decided upon without the constituents of the Hospital—the subscribers—being consulted. (Hear, hear.) It was admitted on every hand that the Hampstead medical men had served the Hospital and the public well. (Hear, hear.) Why the proposed change? Certainly not because there was any need for a change in the medical staff. It was simply that the King's Fund, in the negotiations that had taken place, had arbitrarily demanded that it should be made, and the Council had weakly yielded to their demands. (Hear, hear.) The King's Fund said the great London hospitals had consulted, and therefore this must be. But this was not a great London district where the great hospitals stood, surrounded by heterogeneous masses of the people. The consultant was by no means a fixed quantity; nor was he by any means a sacred quality. There were consultants of the first-class and others of a fifth and sixth-rate order. Some of the medical men attending the Hospital had higher qualifications than some of the consultants of the North-West London Hospital, whom it was proposed—three at least—should be drafted to that Hospital. In conclusion he urged that the first of the resolutions standing in his name was in precise accordance with the views of the Council of the Hospital as set forth in a resolution passed by them on February 23 last. Alderman Fleetwood Pritchard seconded the resolutions.

Mr. W. F. Malcolm (Treasurer of the Hospital) said they had struggled on for twenty years, always in pecuniary difficulties. If it was decided to break off from their engagement with the King's Fund, he was morally certain that within six months they would find it impossible to carry on the Hospital. (Cries of "No.")

Dr. Coode Adams said the financial aspect appeared to have loomed extremely large before the subscribers in the consideration of the matter. The King's Fund had been held up to them as the golden image they were to fall down and worship, but he maintained that Hampstead was perfectly capable of providing the extra £1,000 a year in donations which it had been said would be perfectly sufficient to recoup the Hospital for the loss of the King's Fund.

Eventually Alderman Donald McMillan pointed out the danger of a resolution hostile to the Council being passed, and the desirability of unanimity. He moved an amendment in favour of the appointment of a small committee to consider the question, with power to confer with the Council of the British Medical Association and the Committee of the King's Fund, to report to a meeting to be called at a later date. The resolutions being, by leave, withdrawn for the purpose of facilitating such an inquiry, this amendment was seconded by Mr. Gard and carried almost unanimously, the committee elected being Mr. Walter Baily, Mr. F. Debenham, Mr. H. A. Harben, Miss Clarke, and Mr. Donald McMillan.

Mr. Collins's statement, in reply to a question—before the passing of the amendment—that meantime he would do all he could on the Council "not to take any more definite steps than were absolutely necessary," but that he could not bind the Council, gave some dissatisfaction; but one speaker remarked, amid general approval, that though Mr. Collins was not bound in law they could all be bound in honour, and thereupon the meeting proceeded to the vote, with the result above stated.

